# Genome-wide identification of *MAPK* family in papaya (*Carica papaya*) and their involvement in fruit postharvest ripening

**DOI:** 10.1186/s12870-024-04742-0

**Published:** 2024-01-24

**Authors:** Shengnan Zhu, Yuxing Mo, Yuyao Yang, Shiqi Liang, Shuqi Xian, Zixin Deng, Miaoyu Zhao, Shuyi Liu, Kaidong Liu

**Affiliations:** Life Science and Technology School, Lingnan Normal University, Zhanjiang, 524048 People’s Republic of China

**Keywords:** Mitogen-activated protein kinase (MAPK), Genome-wide analysis, Papaya, Gene expression, Fruit ripening, Ethephon, 1-methylcyclopropene (1-MCP)

## Abstract

**Background:**

Papaya (*Carica papaya*) is an economically important fruit cultivated in the tropical and subtropical regions of China. However, the rapid softening rate after postharvest leads to a short shelf-life and considerable economic losses. Accordingly, understanding the mechanisms underlying fruit postharvest softening will be a reasonable way to maintain fruit quality and extend its shelf-life.

**Results:**

Mitogen-activated protein kinases (MAPKs) are conserved and play essential roles in response to biotic and abiotic stresses. However, the *MAPK* family remain poorly studied in papaya. Here, a total of nine putative *CpMAPK* members were identified within papaya genome, and a comprehensive genome-wide characterization of the *CpMAPKs* was performed, including evolutionary relationships, conserved domains, gene structures, chromosomal locations, *cis*-regulatory elements and expression profiles in response to phytohormone and antioxidant organic compound treatments during fruit postharvest ripening. Our findings showed that nearly all CpMAPKs harbored the conserved P-loop, C-loop and activation loop domains. Phylogenetic analysis showed that CpMAPK members could be categorized into four groups (A-D), with the members within the same groups displaying high similarity in protein domains and intron–exon organizations. Moreover, a number of *cis*-acting elements related to hormone signaling, circadian rhythm, or low-temperature stresses were identified in the promoters of *CpMAPKs*. Notably, gene expression profiles demonstrated that *CpMAPKs* exhibited various responses to 2-chloroethylphosphonic acid (ethephon), 1-methylcyclopropene (1-MCP) and the combined ascorbic acid (AsA) and chitosan (CTS) treatments during papaya postharvest ripening. Among them, both *CpMAPK9* and *CpMAPK20* displayed significant induction in papaya flesh by ethephon treatment, and were pronounced inhibition after AsA and CTS treatments at 16 d compared to those of natural ripening control, suggesting that they potentially involve in fruit postharvest ripening through ethylene signaling pathway or modulating cell wall metabolism.

**Conclusion:**

This study will provide some valuable insights into future functional characterization of CpMAPKs, and hold great potential for further understanding the molecular mechanisms underlying papaya fruit postharvest ripening.

**Supplementary Information:**

The online version contains supplementary material available at 10.1186/s12870-024-04742-0.

## Background

Plants, being sessile organisms, frequently encounter a plethora of abiotic and biotic stresses during growth and development processes, including pathogen infection, drought, salinity, extreme temperature and nutrient deficiencies, that threaten their survival [1–3]. To counter the detrimental environmental stimuli and in pursuit of optimal growth conditions, plants have evolved a series of intricate signaling mechanisms to cope with and adapt to these challenges [3, 4]. Plants respond to environmental stimuli by modulating the expression of numerous genes or changing protein activity through transcriptional regulation or post-translational modifications (PTMs), such as phosphorylation, SUMOylation, acetylation and ubiquitylation modifications [1, 5, 6]. Protein phosphorylation, as one of the most important and ubiquitous PTMs, is orchestrated by a large number of protein kinase families, such as calcium-dependent kinases (CDPKs), sucrose nonfermenting 1 (SNF1), mitogen-activated protein kinase (MAPK) cascades, and receptor-like kinase (RLK) families [6–8]. As the archetypal signaling modules, MAPK cascades have been identified to participate in a series of biological processes by relaying the external stimuli into cellular response [9, 10].

A typical MAPK cascade encompasses three functionally interlinked protein kinases, namely MAPKK kinase (MAPKKK), MAPK kinase (MAPKK) and MAPK, all of which act as sequential signal transducers to regulate the activities and stabilities of specific target proteins via phospho-relay system [11–13]. MAPK, as the terminal player of MAPK signaling cascades, serine and tyrosine residues in the TXY motifs which are sequentially phosphorylated by MAPKK, exhibit more complexity and sequence diversity in plants [9, 10]. Recently, a large number of MAPK genes were identified in plants with the availability of plant genome and transcriptome sequencing, such as 20 members in Arabidopsis (*Arabidopsis thaliana*) [14], 15 in rice (*Oryza sativa*) [15], 19 in maize (*Zea mays*) [16], 43 in cultivated strawberry (*Fragaria ananassa*) [17], 18 in kiwifruit (*Actinidia Chinensis*) [18], 26 in apple [19] (*Malus domestica*), 14 in cucumber (*Cucumis sativus*) [20] and 25 in banana (*Musa acuminata*) [21]. Accumulating evidence have demonstrated that plant MAPKs could be divided into four subgroups, designated as group A, B, C and D, on the basis of their activation loop TX(D/E)Y motifs and evolutionary relationships [22–24]. MAPKs within group A-C contained a T-E-Y motif in their phosphorylation sites, while those in the group D had a T-D-Y motif in their activation loops [25, 26].

Numerous studies have elucidated the pivotal roles played by MAPKs in the regulation of biotic and abiotic stresses [9, 26]. For instance, rice MAPK33 was found to exert a negative regulatory effect on plant salt tolerance by suppressing the expression of genes involved in the K^+^/Na^+^ iron transport [27]. Unlike OsMAPK33, OsMAPK5 was demonstrated to act as a positive regulator of salt tolerance [28]. Additionally, the activation of heat shock factor A4A (HSFA4A) by MAPK3/4/6 has been reported to enhance Arabidopsis heat and salt tolerance by reducing the oxidative damage [29]. In addition, MAPKs have also been implicated in hormone signaling pathways, where they are individually or collaboratively activated to regulate several rapid responses, such as stomatal closure and gene expression [13, 30]. For example, the MAPK3K17/18-MKK3-MAPK1/2/7/14 pathway in Arabidopsis is primarily activated by the ABA core module in response to drought stress, subsequently enhancing the transcript abundances of a series of osmotic-responsive or drought-related genes [13, 26]. Moreover, the rice polar auxin transport (PAT) proteins can undergo reversible phosphorylation by MAPKs and other phosphatases, thereby controlling auxin transport [31].

Beyond their roles in biotic and abiotic stress responses, some MAPKs have been proposed to play crucial roles in plant development and fruit ripening [7, 32–34]. For example, it has been observed that most of the *FvMAPK* genes exhibit higher expression levels during fruit development stages in cultivated strawberry [17]. In banana, MAPK6-3 has been reported to act as a positive regulator that phosphorylates bZIP21 to increase the transcriptional activation of its downstream ripening-associated genes, thus promoting fruit ripening [34]. Similarly, several studies have demonstrated that the MaMAPK2-MabZIP93 module enhances the fruit ripening by mediating the transcriptional reprogramming of cell wall-modifying genes including *pectin esterase 1* (*MaPE1*) and *pectate lyase* (*MaPL2*), while MaMAPK11-3-MabZIP74 module plays essential roles in directly regulating the expression of two 1-aminocyclopropane-1-carboxylicacid oxidase-encoding genes, *ACO1/4* [33, 34]. Moreover, emerging evidence has unveiled that CsMAPK6-mediated phosphorylation of CsMYC2 represses the expression of fruit coloration-related genes via regulating jasmonate signaling pathway [35]. These collective findings imply that MAPK proteins may indirectly function in fruit ripening by modulating the activities of transcription factors or other proteins.

Papaya (*Carica papaya*) is an important fleshy fruit with both nutritional and pharmacological properties due to its compositions, including vitamin C, minerals and phenolic compounds [36, 37]. Being a climacteric fruit, papaya fruit has a short shelf-life attributing to its rapid ripening and softening after postharvest, thus leading to a consequent decrease in fruit quality [38, 39]. Although some genes and proteins have been identified to be associated with papaya fruit ripening, including transcription factors belonging to ethylene response factor (ERF), auxin response factor (ARF), basic-helix-loop-helix (bHLH) transcription factors, and NAM-ATAF1/2-CUC2 (NAC) families [36, 38, 40, 41], the roles of CpMAPKs in mediating fruit ripening in papaya remain largely unexplored.

In this study, we conducted a comprehensive study on gene structures, chromosomal locations, promoter analysis and evolutionary relationships of MAPKs. Moreover, the expression levels of MAPKs in response to ethephon, 1-MCP and the combined AsA and chitosan treatments at different fruit postharvest ripening stages were analyzed by qRT-PCR, and several candidate genes related to fruit ripening were also identified. These results will lay the foundations for elucidating the roles of MAPKs in regulation of papaya fruit postharvest ripening.

## Results

### Identification and characterization of MAPK family members in papaya

A total of nine putative MAPK protein-encoding genes were identified in papaya genome, which were annotated in terms of their homologs in Arabidopsis, designated as *CpMAPK3/4/6/7/9/13/17/19/20* (Table 1). The general information of MAPK family is summarized in Table 1. It was obvious that the lengths of ORFs ranged from 1,113 to 1,893 bp, corresponding to the encoded proteins with molecular weights varying from 43 to 72 kD (Table 1). In addition, five out of nine CpMAPK proteins exhibited basic characteristics, with isoelectric point (*pI*) values being over 7.0, while the remaining four members displayed acid properties (Table 1). Moreover, none of these CpMAPK proteins had transmembrane domains in their *N* or *C* terminals. Subcellular localization analysis showed that all CpMAPKs were localized in the nucleus (Table 1).

**Table 1:** General information on MAPK gene family in papaya

Proposed name	Accession number	Gene ID	chromosomal location	Strand^a^	Length of ORF (bp)	Mw^b^ (kD)	*pI*^c^	TM^d^ domain	T-loop type	Subcellular localization	Homologous gene
*CpMAPK4*	XP_021887822	evm.TU.supercontig_184.17	supercontig_184:162,606..170057	-	1125	43	6.24	No	TEY	Nucleus	AtMAPK4
*CpMAPK13*	XP_021889803	evm.TU.supercontig_139.35	supercontig_139:214,725..218018	-	1122	43	5.02	No	TEY	Nucleus	AtMAPK13
*CpMAPK3*	XP_021889758	evm.TU.supercontig_139.47	supercontig_139:358,399..360860	-	1113	43	5.6	No	TEY	Nucleus	AtMAPK3
*CpMAPK6*	XP_011199890	evm.TU.supercontig_343.2	supercontig_343:5871..10478	-	1218	46	5.34	No	TEY	Nucleus	AtMAPK6
*CpMAPK7*	XP_021907914	evm.TU.supercontig_6.174	supercontig_6:1,435,616..1439341	+	1119	43	7.6	No	TEY	Nucleus	AtMAPK7
*CpMAPK9*	XP_021909450	evm.TU.supercontig_3.416	supercontig_3:2,948,540..2955310	+	1659	62	8.3	No	TDY	Nucleus	AtMAPK9
*CpMAPK17*	XP_021896299	evm.TU.supercontig_65.147	supercontig_65:1,291,730..1295805	-	1593	61	8.96	No	TDY	Nucleus	AtMAPK17
*CpMAPK19*	XP_021911774	evm.TU.contig_29215	contig_29215:6300..10777	-	1794	68	9.27	No	TDY	Nucleus	AtMAPK19
*CpMAPK20*	XP_021898364	evm.TU.supercontig_50.81	supercontig_50:803,301..809482	-	1893	72	9.03	No	TDY	Nucleus	AtMAPK20

^a^Strand direction, ‘-’ or ‘ + ’ indicates reverse or forward strand, respectively

^b^Mw, molecular weight

^c^pI, isoelectric point

^d^TM, transmembrane domain

### Multiple sequence alignments and phylogenetic analysis

**Fig. 1:**
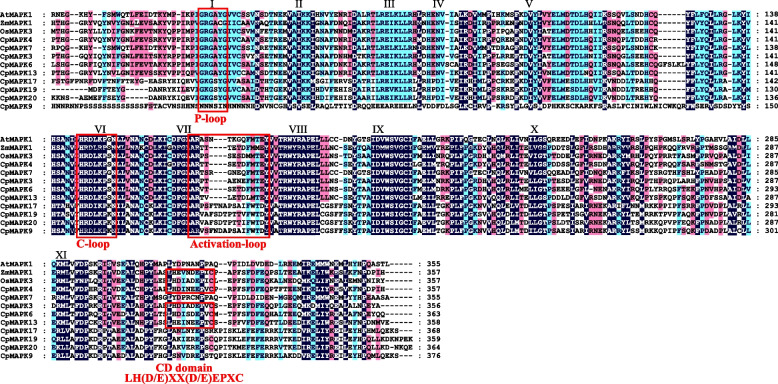
Multiple sequence alignment of the conserved kinase domains of CpMAPKs. The multiple alignment is conducted using ClustalX, and is presented using GeneDoc. The eleven conserved kinase domains that have been found among plant MAPKs are indicated by *Roman numerals* (I to XI). The conserved loop domains, including P-loop, C-loop, activation loop and CD-domain, are shown in red boxes. The first two letters of each MAPK indicate the abbreviated species name. At: *Arabidopsis thaliana*; Zm: *Zea mays*; Os: *Oryza sativa*; Cp: *Carica papaya*

Multiple sequence alignments between CpMAPKs and other plant MAPKs were conducted. The results showed that all of the CpMAPK proteins, except for CpMAPK9, contained 11 characteristic and highly conserved kinase subdomains (I-XI), which were closely resembled those found in other plant MAPK proteins, such as AtMAPK1, ZmMAPK1 and OsMAPK3 (Fig. 1). Besides, five out of nine CpMAPK proteins (CpMAPK3/4/6/7/13) were found to possess the T-E-Y motifs, while the remaining four proteins (CpMAPK9/17/19/20) harbored the T-D-Y motifs in the activation loops (Fig. 1). Moreover, we found that the conserved catalytic C-loop (HRDLKP[G/S/K]N) was present in all CpMAPK proteins, while the CD domain (LH[D/E]XX[D/E]EPXC) that acted as the docking site for substrates was observed in four CpMAPK members (CpMAPK3/4/6/13). Meanwhile, eight out of nine CpMAPK proteins were found to possess P-loop motifs (GRG[A/S]YG), serving as the phosphate-binding sites, in their *N*-terminals (Fig. 1).

**Fig. 2:**
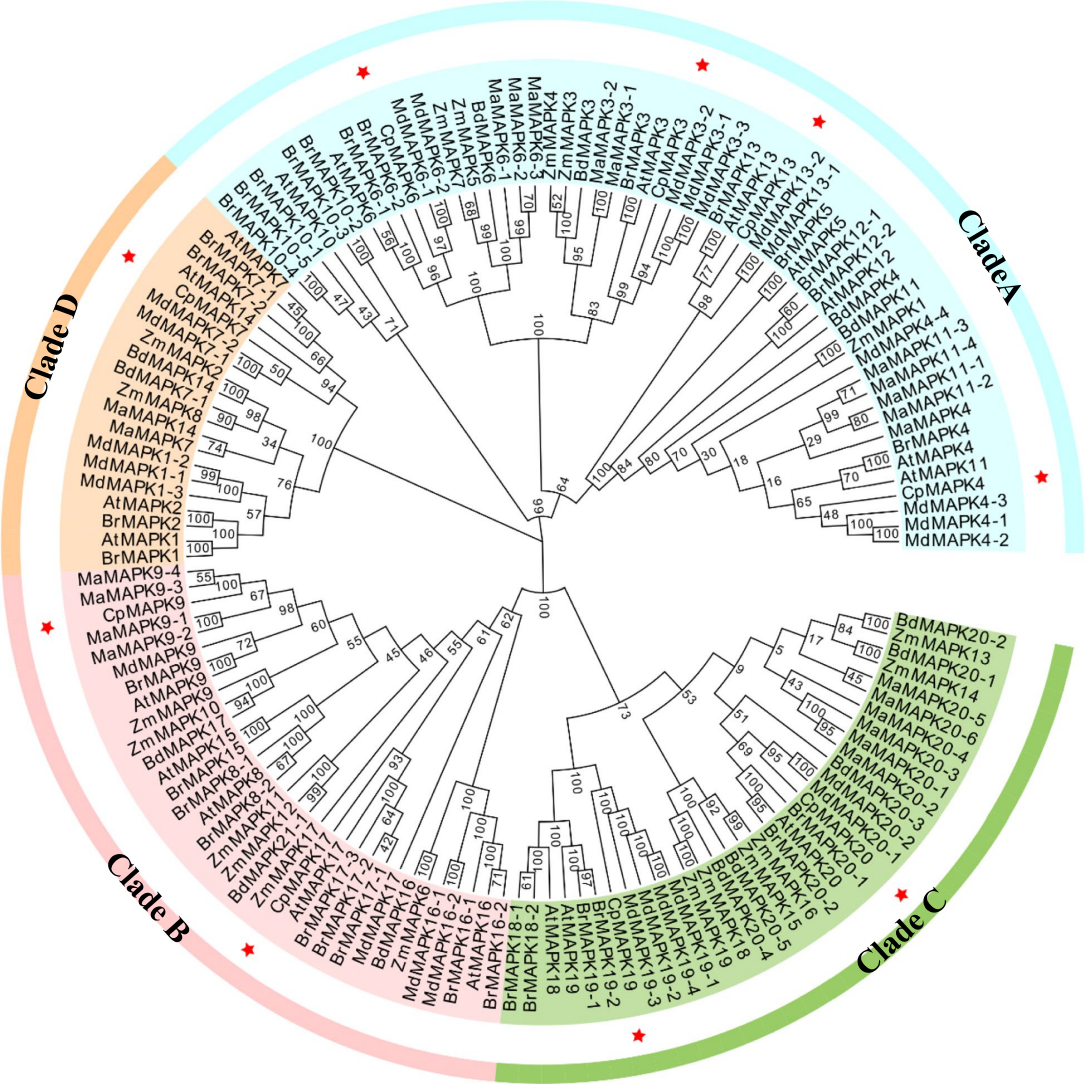
Phylogenetic analysis of MAPK proteins in plants. The phylogenetic tree was constructed using MEGA 11 program with the neighbor-joining method, and bootstrap value is set to 1000, which is indicated as percentages for the branches. The first two letters of each MAPK indicate the abbreviated species name. Zm: *Zea mays*; At: *Arabidopsis thaliana*; Bd: *Brachypodium distachyon*; Ma: *Musa acuminata*; Md: *Malus domestica*; Br: *Brassica rapa*; Cp: *Carica papaya*. The members of CpMAPK family are separately indicated by red stars

To gain insights into the potential roles of CpMAPK proteins, the phylogenetic analysis of 142 MAPKs from different plant species, such as 19 from maize, 20 from Arabidopsis, 26 from apple, 22 from banana, 32 from *B. rapa* (*Brassica rapa*), 14 from purple false brome (*Brachypodium distachyon*) and 9 from papaya were constructed, as depicted in Fig. 2. The results showed that all plant MAPKs could be categorized into four clades (A-D) with uneven CpMAPK members (Fig. 2). Among these, four out of nine MAPKs, namely CpMAPK3, 4, 6 and 13, were distributed into clade A, the largest clade that also contained 51 MAPKs from other species, including 11 MdMAPKs, 10 MaMAPKs, 8 AtMAPKs, 5 ZmMAPKs, and 4 BdMAPKs and 13 BrMAPKs (Fig. 2)*.* Remarkably, CpMAPK6 shared high sequence similarity with MAPK6-3 known to be a positive regulator in involvement of MabZIP6-3-mediated fruit ripening (Fig. 2). Additionally, two CpMAPKs (CpMAPK9 and CpMAPK17), together with 31 MAPK proteins from other plants, were clustered into clade B, while two CpMAPK proteins (CpMAPK19 and CpMAPK20) were belonged to clade C (Fig. 2). Lastly, only one CpMAPK, CpMAPK7, was assigned to clade D, the smallest clade with only 20 MAPK members.

### Gene structure, conserved motif and domain analysis of CpMAPKs

**Fig. 3:**
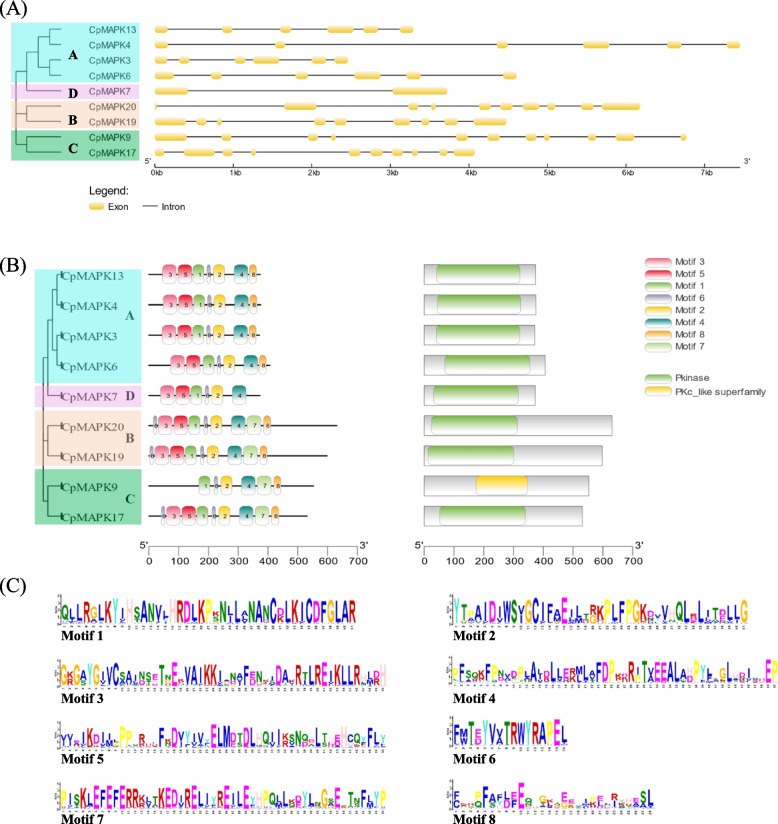
Analysis of gene structures and protein structures of CpMAPKs in papaya. **A** Gene structure analysis of *CpMAPKs*. The exons of *CpMAPKs* were indicated by the yellow boxes, and the introns were indicated by the middle lines. **B** Conserved motif and domain analysis of CpMAPKs. **C** Analysis of conserved amino acids of motifs. The upper case in the left panel indicated the different subgroups of CpMAPKs in phylogenetic tree

Using coding sequences (CDSs) and corresponding genomic sequences, the gene structures of all *CpMAPKs* were constructed and analyzed. The results showed that the number of exons in *CpMAPKs* varied from 2 to 11, along with the number of introns ranging from 1 to 10 (Fig. 3A). Combined with the phylogenetic relationship analysis, we found that the exon–intron organizations were highly conserved in the same group, but exhibited divergence among different subgroups (Fig. 3A). For instance, the CpMAPK members of group A consisted of six exons, whereas those in group B and C had 9 to 11 exons. Strikingly, CpMAPK7 contained only one intron and two exons, the number of which was lesser than those in other members.

To further explore the diversity and evolutionary relationships among CpMAPK members, we analyzed the conserved motifs and domains. The results showed that a total of eight motifs were found amongst the nine CpMAPKs using the MEME program (Fig. 3B). It was obvious that the members in the same group shared similar motif patterns. Moreover, four motifs were present in all of the CpMAPK members (Fig. 3B and C), among which was motif 6, that possessed the typical T(D/E)Y loop (Fig. 3C). Moreover, all CpMAPK members in group B and C had an extra motif 6 in their *N*-terminus, except for CpMAPK9, while the members in group A lacked a specific motif 7 (Fig. 3B). Additionally, the conserved domain analysis showed that all the CpMAPK proteins contained a complete Pkinase domain, apart from CpMAPK9, which only had the C-terminal region of the Pkinase domain (Fig. 3B).

### Promoter region analysis of CpMAPKs

**Fig. 4:**
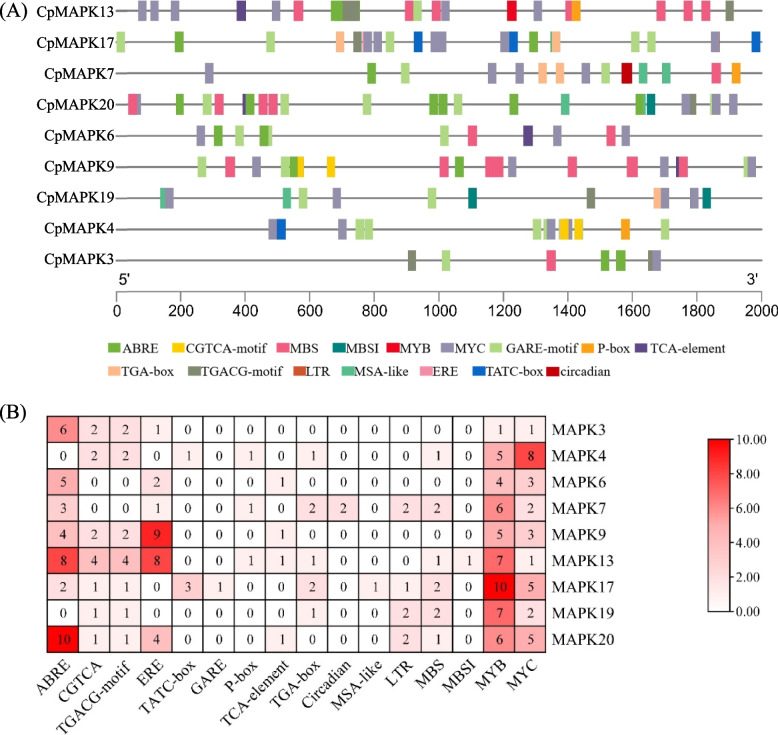
The *Cis*-regulatory elements analysis of *CpMAPK* genes. **A** The distribution of *cis*-elements of *CpMAPKs*. **B** The number of *cis*-elements of *CpMAPKs*. ABRE: abscisic acid-responsive element; CGTCA/TGACG-motif: MeJA-responsive elements; MYB/MBS/MBSI: MYB transcription factor binding sites; MYC: MYC transcription factors binding sites; TATC-box/GARE-motif/P-box: gibberellin-responsive element; TCA-element: salicylic acid-responsive element; TGA-box: auxin-responsive element; LTR: low-temperature-responsive element; MSA-like: cell cycle regulation; ERE: ethylene-responsive element; Circadian: circadian control

To further understand the gene function and transcriptional regulation of *CpMAPK* genes, 2.0-kb genomic DNA sequences upstream of the start codons were used to analyze the *cis*-elements, as shown in Fig. 4. A total of 16 different types of *cis*-elements were listed in promoter regions of CpMAPK genes, which could be categorized into three categories. The first category consisted of hormone-related elements, including auxin-responsive (TGA-box), ethylene-responsive (ERE element), abscisic acid-responsive (ABRE motif), salicylic acid-responsive (TCA-element), methyl jasmonate (MeJA)-responsive (CGTCA, TGACG-motif) and gibberellin-responsive (TATC-box/GARE-motif/P-box) elements. These elements were found in the promoters of CpMAPK genes, with a notable enrichment of ERE- and ABRE-elements. The second category was identified as stress response-related elements, such as the low-temperature-response element (LTR), observed in four *CpMAPK* (7, 17, 19 and 20) genes (Fig. 4A, B). Additionally, MYB (i.e., MYB, MBS, and MBSI) and MYC transcription factor binding sites were identified in all MAPKs (Fig. 4). Moreover, cell cycle regulation and circadian elements, belonging to the plant growth and development category, were separately observed in the promoters of *CpMAPK17* and *CpMAPK7*.

### Expression analysis of CpMAPKs in response to hormone treatments

**Fig. 5:**
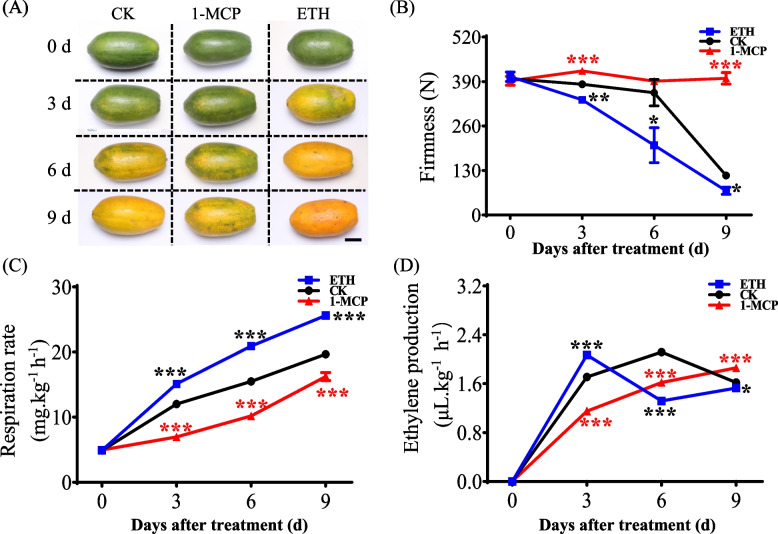
Effects of 1-MCP and ethephon on fruit firmness, respiration rate and ethylene production during papaya fruit postharvest ripening. **A** Appearance of papaya fruits during natural, 1-MCP-delayed and ethephon-induced ripening. **B** Changes in firmness. **C** Respiration rate. **D** Ethylene production rate. The green-mature papaya fruits were harvested, and then separately treated with 0.5 g/L ethephon for 5 min, and 0.5 μL/L 1-MCP for 2 h. The untreated fruits were used as a control. Each experiment was conducted with three biological replicates, and each replicate with three fruits. The red asterisks indicate significant differences between CK and 1-MCP treatments, and black asterisks indicate significant differences between CK and ethephon treatments by Student’s* t*-test: * *P* < 0.05; ** *P* < 0.01; *** *P* < 0.001, respectively

To explore the effects of hormone treatments on papaya fruit postharvest ripening, the uniform green-mature papaya fruits were harvested and treated with 1-MCP and ethephon-treatments, respectively. During storage, 1-MCP treatment significantly inhibited the fruit ripening, as reflected by the delayed yellowing, the higher firmness as well as the lower respiration rate (Fig. 5A-C). In contrast, the respiration rate was dramatically higher in ethephon-treated fruits compared to those under natural ripening conditions, which was in accordance with the reduced firmness of the papaya fruits exposed to ethephon treatment compared to natural ripening fruits (Fig. 5B, C). Moreover, the ethylene production peaked at 6 d of the natural ripening controls, while the peak of it separately came out at 3 d under ethephon treatment, and 9 d and 1-MCP treatment (Fig. 5D).

**Fig. 6:**
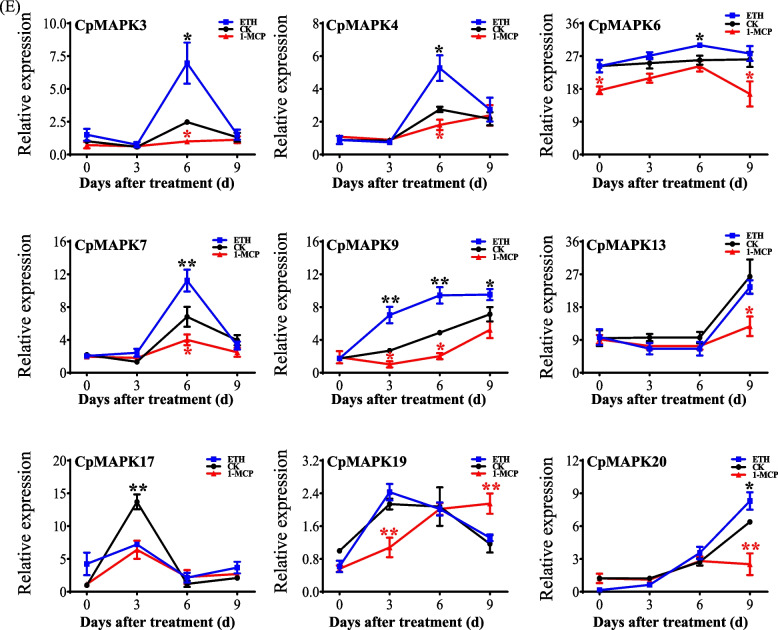
The expression levels of *CpMAPKs* in fruit flesh in response to ethephon (ETH) and 1-MCP treatment. The papaya flesh tissues were sampled at 0, 3, 6 and 9 d after being subjected to ethephon and 1-MCP treatment. The relative expression levels were determined by qRT-PCR, and calculated by ΔΔ*C*_t_ method. The red asterisks indicate significant differences between CK and 1-MCP treatments, and black asterisks indicate significant differences between CK and ethephon treatments by Student’s* t*-test: * *P* < 0.05; ** *P* < 0.01, respectively

To further elucidate the roles of *CpMAPKs*, the expression of *CpMAPKs* in the peel and flesh tissues were analyzed under different hormone treatments during fruit different postharvest stages (Fig. 6). Our results showed that the transcripts of all nine CpMAPK members were detectable in fruit flesh and peel tissues during postharvest ripening stages, except for those of two members in peel (i.e., CpMAPK6 and CpMAPK9), as shown in Fig. 6 and Fig. S1. All nine *CpMAPKs* displayed various expression patterns in response to ethephon and 1-MCP treatments during postharvest ripening stages. Compared to those under normal controls, the expression levels of three CpMAPKs, namely *CpMAPK3, 4* and *7*, were initially no distinction in fruit flesh tissues for the first three days under ethephon and 1-MCP treatments, but the pronounced inductions of them were 2.8-, 1.9- and 1.6-fold by ethephon treatment at 6 d, respectively (Fig. 6). Moreover, transcripts of them in fruit flesh tissues were inhibited by 1-MCP treatments, as reflected by 60%, 34% and 41% decreases, respectively, at 6 d (Fig. 6). In contrast, the abundance of both *CpMAPK13* and *CpMAPK20* in fruit flesh was not influenced by 1-MCP and ethephon addition at the early 6 d treatment, whereas both of them were down-regulated at 9 d, as reflected by 52% and 60% decreases under 1-MCP treatment, irrespective of a 32% increase of CpMAPK20 after 9-d ethephon treatment (Fig. 6). Besides, the expression of *CpMAPK9* exhibited a constantly significant increase with the increased duration of postharvest ripening stages in all treatments, where *CpMAPK9* was found to be up-regulated by ethephon treatment at the whole postharvest stages, and be inhibited by 1-MCP treatment at the 3 d and 6 d, respectively (Fig. 6). Interestingly, *CpMAPK19* exhibited a specific response to hormone treatment, with displaying an increasing trend under 1-MCP treatment during fruit postharvest ripening, but an opposite trend in response to ethephon-treatment and normal postharvest ripening. It is worth noting that the expression level of *CpMAPK19* remained unaffected by ethephon application (Fig. 6). Furthermore, the *CpMAPK17* transcript abundance in flesh was separately decreased after exogenous ethephon and 1-MCP treatment at 3 d, being around 47–53% lower than that under natural ripening conditions. In addition, the expression level of *CpMAPK6* exhibited an increase at 6 d under ethephon treatment, but was inhibited at 0 d and 9 d by 1-MCP treatment, respectively (Fig. 6).

In addition, the expression patterns of the detected *CpMAPKs* were highly similar in response to hormone treatments at various postharvest stages (Fig. S1). Among these, the expression levels of three *CpMAPK* members (*CpMAPK13*, *3* and *20*) in peel were gradually decreased with the increased duration of ethephon and 1-MCP treatments. Moreover, the transcripts of *CpMAPK3* in the peel at 0 d under both ethephon and 1-MCP treatment were enhanced by 50% compared to those observed under CK normal ripening, while the expression levels of *CpMAPK20* exhibited no difference between these treatments (Fig. S1). Conversely, three other *CpMAPKs* (*CpMAPK7*, *17* and *19*) exhibited increased expression levels within prolonged duration of all treatments. Notably, the transcripts of CpMAPK17 in the peel were down-regulated under both ethephon and 1-MCP treatment at 6 d, while the expression of CpMAPK19 was separately enhanced by ethephon application at 3 d and 1-MCP application at 9 d (Fig. S1).

### Expression analysis of CpMAPKs in response to the combined AsA and chitosan treatments

**Fig. 7:**
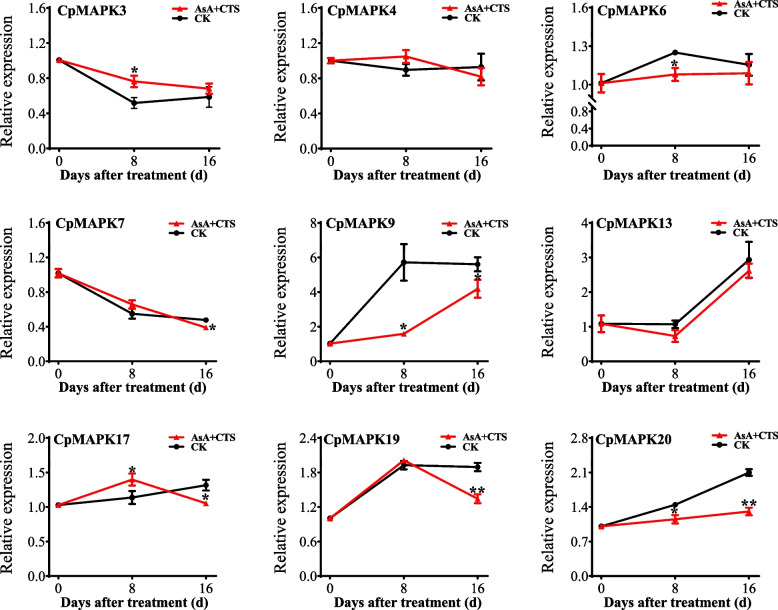
The expression levels of *CpMAPKs* in papaya flesh in response to the combined treatment of AsA and chitosan. The papaya fruit tissues were sampled at 0, 8 and 16 d after being subjected to AsA and CTS treatment. Untreated fruits were used as a control. The relative expression levels were determined by qRT-PCR, and calculated by ΔΔ*C*_t_ method. The asterisks indicate significant differences between CK and AsA + CTS combined treatments by Student’s* t*-test: * *P* < 0.05; ** *P* < 0.01, respectively

Our team have found that the combined treatments of AsA and chitosan have delayed the papaya fruit ripening by scavenging the reactive oxygen species generated during senescence and modulating the genes associated with cell wall metabolism [39]. However, whether CpMAPKs participate in the combined effects of AsA and CTS on fruit postharvest ripening remain obscure. In this study, the expression patterns of *CpMAPKs* in papaya flesh in response to the combined treatment of AsA and CTS at different postharvest stages (8 d and 16 d) were investigated with quantitative real-time PCR (qRT-PCR). The expression levels differed with *CpMAPKs*, but the addition of exogenous AsA and CTS indeed resulted in the down-regulation of almost all *CpMAPKs* in papaya flesh at 8 d or 16 d, except for *CpMAPK4* and *CpMAPK13* (Fig. 7). In contrast, the expression levels of *CpMAPK4* and *CpMAPK13* was separately not affected by AsA and CTS additions (Fig. 7). Collectively, all results suggested that *CpMAPKs* might play important roles in the fruit postharvest ripening.

## Discussion

As important sources of bioactive compounds and nutrients, fruits are indispensable in human’s daily diets [42]. Ripening of fleshy fruits involved a series of sophisticated developmental, hormonal and epigenetic regulations as well as environmental factors, which acted individually or coordinately to alter the postharvest quality attributes, such as aroma, flavor, texture and color [43, 44]. Among them, the involvement of transcriptional regulation in fruit ripening is mediated by a variety of transcription factors, including *NACs*, *MYBs*, basic helix-loop-helix proteins (*bHLHs*) and *ARF*s [34, 38, 45]. Although the functions of key TFs or proteins in fruit ripening have been extensively investigated, however, the roles of post-translational phosphorylation modifications in regulation of TFs remain largely unknown.

MAPKs belonging to the serine/threonine-protein kinase family, have been well studied in many plant species, such as Arabidopsis, maize and rice [14, 18, 21]. However, there is little knowledge on the roles of the MAPK family in papaya. In this study, a total of nine *CpMAPK* genes were identified in papaya, the family size of which is smaller than the number of members identified in other plant species [14, 15, 18, 21, 46]. Phylogenetic analysis showed that nine CpMAPKs could be divided into four subgroups (Fig. 2), which is consistent with the findings in other studies [14, 15]. Remarkably, five out of nine CpMAPK proteins (CpMPK3/4/6/7/13) contained the T-E-Y motif, while the remaining four members exhibited the T-D-Y motif (Fig. 1). This conservation of motifs aligns with observations in various plants, such as Arabidopsis, tomato, apple, grape and strawberry, suggesting that the MAPK members in plants are relatively conserved during the courses of evolution [47]. Moreover, the members of CpMAPK family were unevenly clustered into four clades in the phylogenetic tree (Fig. 2). Among them, CpMAPK6 shared high sequence similarity with AtMAPK6 and MaMAPK6 (Fig. 2). In Arabidopsis, AtMAPK6 was known to phosphorylate AtERF72, and thus enhance its DNA binding activity to modulate defense signaling in Arabidopsis [48]. Similarly, banana MaMAPK6-3 has been reported to phosphorylate MabZIP21, thus enhancing its transcriptional activation ability to regulate the expression of a subset of ripening-associated genes [8]. In addition, CpMAPK3 together with MdMAPK3 (3–1, 3–2, 3–3) and AtMAPK3 were grouped into clade I, where MdMAPK3 was known to play an important role in MAKK4-MAPK3-WRKY17-mediated salicylic acid degradation during apple leaf virus infection [49]. Therefore, it is plausible that CpMAPKs may serve similar functions to their closest MAPK homologs from other plant species, which merits further analysis.

To investigate the expression patterns of *CpMAPKs* in response to 1-MCP and ethephon treatments at distinct fruit postharvest ripening stages, the papaya fruits were subjected to different hormone treatments after postharvest (Figs. 5 and 6). It was observed that the fruit peel color turned quickly from green to yellow with increased duration of ethephon treatment compared to that under natural ripening conditions. Moreover, the application of 1-MCP had delayed the fruit ripening, which was consistent with the changes of firmness in Fig. 5B. Meanwhile, the qRT-PCR analysis showed that the expression patterns of *CpMAPKs* differed from each other. Consistently, similar findings have been reported for 25 *MaMPKs* in banana, which also exhibited diverse responses to ethephon and 1-MCP treatments [21]. Of the detected nine *CpMAPK* genes, five *CpMAPKs* were significantly up-regulated under ethephon treatment at 6-d (Fig. 6), which was consistent with the enhanced expression levels of *MaMAPK6-3* during ethylene-induced ripening [21]. Emerging evidence suggested that transient overexpression of *MaMPK6-4* in bananas significantly promotes the fruit ripening by increasing the abundance of phosphoproteins in transgenic materials [8]. These results suggesting that the five ethephon-induced *CpMAPKs* (*CpMAPK3*, *4*,* 6*, *7* and *9*) might play positive roles in papaya fruit ripening. In contrast, previous studies have demonstrated that *MaMAPK14* transcript exhibited a significant decrease with the increased ripening time, and overexpression of *MaMAPK14* showed lesser ethylene production, and higher fruit firmness compared to control lines [50]. In addition, the transcripts of four *FaMAPKs* (*FaMAPK3-3*, *7–4*, *16–1* and *20–1*) were significantly decreased from the initial-red stages to the full-red stages [17]. Interestingly, *CpMAPK17* was significantly inhibited by both ethephon and 1-MCP treatment in flesh tissues at 3 d after postharvest (Fig. 6), suggesting that a novel and different regulatory role of CpMAPK17 might exist, which might exert in papaya fruit ripening.

Emerging evidence showed that a series of effective strategies have been developed to delay the fruit postharvest ripening and prolong the shelf-life of fruits, including heat treatment, hydrogen-rich water, cold storage and edible coating [39, 51, 52]. In previous studies, our team has found that the edible chitosan coating plays an important role in maintaining fruit quality by improving the antioxidant capacity [39]. On this basis, the expression levels of *CpMAPKs* in response to the combined treatment of AsA and chitosan were investigated (Fig. 7). It was obvious that six out of nine *CpMAPKs* (i.e., *CpMAPK3*, *6*, *9*, *17*, *19* and *20*) were down-regulated after addition of exogenous AsA and chitosan, which was consistent with the previous one showing that most *CpMAPK* was inhibited in delayed ripening fruits under 1-MCP treatment as shown in Fig. 5. Therefore, it is further demonstrated that CpMAPK might play different roles in fruit postharvest ripening, which merits further functional analysis.

## Conclusions

In summary, our study identified a total of nine *CpMAPK* members in the papaya genome. We conducted a comprehensive genome-wide analysis of the CpMAPKs, including multiple sequence alignment, phylogenetic relationships, conserved domains, chromosome localization, *cis*-regulatory elements and expression patterns in response to ethephon, 1-MCP and the combined treatment of AsA and CTS. The expression profiles suggest that *CpMAPK* genes may be involved in fruit ripening by modulating the activities and stabilities of several specific target proteins. This study not only provides valuable insights into the mechanisms underlying papaya fruit ripening, but also offers some strategies for the genetic improvement of fruit with high quality.

## Materials and methods

### Plant materials and treatments

Green-mature papaya (*Carica papaya* L. cv. ‘Daqing’) fruits with maturity degree of 70–80% (the peel color < 10% yellow) were purchased from a local farm in Zhanjiang, Guangdong, China, as described previously [38, 53, 54]. Immediately after harvest, the uniform papaya fruits without any visible injuries were sterilized with 1% (v/v) sodium hypochlorite (NaClO) for 5 min, followed by being air-dried overnight. For 1-MCP and ethephon treatments, the cleaned fruits were divided into three sets. The first set was immersed with 0.5 g/L ethephon (Macklin, China) solution for 5 min, and the second one was treated with 0.5 μL/L 1-MCP for 2 h, as previously described with slight modifications [8, 55]. The remaining one was left untreated, and underwent natural ripening. Following treatments, the fruits were stored at room temperature (around 22 ~ 24℃) and 70% relative humidity for a period time. During this storage period of time, fruit samples, including both peel and flesh, were separately collected from each treatment at the given sampling points (0 d, 3 d, 6 d and 9 d), and immediately quickly frozen in liquid nitrogen and stored at -80℃ for further use. Three biological replicates of nine fruits were conducted for each sampling point (3 fruits constituted 1 replicate).

In the previous study, our team found that AsA and chitosan CTS combined treatment could maintain the postharvest quality of papaya fruits [39]. To investigate the response of *CpMAPKs* to combined treatment of AsA and CTS, the picked papaya fruits were sprayed with the mixed solution of 1.5% (w/v) AsA and 1.0% (v/v) CTS during storage, and the fruit flesh was sampled every 8 d, as previously described [39]. Moreover, the picked fruits treated with deionized water were used as a control. Three biological replicates with three fruits each were conducted.

### Fruit ripening measurement

The ripening indexes of papaya fruits, including firmness, ethylene production and respiration rate, were measured according to methods from previous studies [38, 50]. As for firmness measurement, the fruits were penetrated using an 8-mm cylindrical flat-surfaced plunger that was equipped with a penetrometer (model no.5542; Instron, Canton, MA, USA). The firmness was recorded in three different fruits, with three different points per fruit. The results were expressed as Newton (N).

For ethylene production, harvested fruit samples were collected into 5-mL airtight glass bottles at 26℃ for 3 h. Subsequently, 1 mL of air was withdrawn from the bottles, and injected into a gas chromatograph (Shimadzu Corp., Kyoto, Japan) fitted with an activated alumina column and a flame ionization detector.

### Genome-wide identification of MAPK family in papaya

To identify the MAPK family in papaya, both BLASTP and Hidden Markov Model (HMM) searches (http://hmmer.janelia.org/) were performed using MAPK proteins from Arabidopsis that were retrieved from Phytozome (https://phytozome-next.jgi.doe.gov/) database [14]. All the putative papaya MAPK protein sequences were downloaded from Phytozome database, and screened by using NCBI-CDD (https://www.ncbi.nlm.nih.gov/cdd) and Smart domain analysis (http://smart.embl.de/smart/set_mode.cgi?NORMAL=1) to filter the redundant candidate proteins lacking the typical structural domains or features, as described previously [8, 47]. Besides, the biochemical and molecular characterization of MAPKs, such as chromosomal locations, open reading frames (ORFs) and amino acid numbers, protein molecular weight (MW), exon/intron structure, and isoelectric point (*pI*) were separately retrieved and performed by using ExPASy ProtParam tool (https://web.expasy.org/protparam/) and Phytozome database, as previously described [55]. Moreover, the subcellular locations of CpMAPK proteins were predicted using Plant-mPLoc website (http://www.csbio.sjtu.edu.cn/bioinf/plant-multi/), and the conserved motif analysis of CpMAPK proteins were conducted using the MEME program (https://meme-suite.org/meme/tools/meme).

### Multiple sequence alignments and phylogenetic tree analysis

The multiple sequence alignments of the CpMAPK family, and AtMAPK1 (accession No. NP_001031017) from Arabidopsis [14], OsMAPK3 (accession No. NP_001389066) from rice [15] and ZmMAPK1 (accession No. GRMZM2G123886_P01) from maize [37] were carried out using ClustalX 2.1 software with the default setting parameters. The alignment results were visualized using GeneDoc software [47]. Additionally, the phylogenetic tree of plant MAPKs was constructed by MEGA 11.0 using the neighbor-joining (NJ) method with 1000 bootstrap values, and the tree was visualized and ornamented using Evolview (http://www.evolgenius.info/evolview/#/).

### *Cis*-regulatory element analysis of CpMAPKs

Using CDSs and corresponding genomic sequences, the exon/intron organizations of all MAPK genes in papaya were obtained using Gene Structure Display Server 2.0 (http://gsds.gao-lab.org/). To investigate *cis*-elements present in promoter regions of MAPK genes, the 2.0-kb upstream sequences from the start codon were downloaded and submitted to the PlantCARE database (http://bioinformatics.psb.ugent.be/webtools/plantcare/html/) to predict the putative *cis*-elements. The identified elements within the promoter regions were visualized using the TBtools software [43].

### RNA extraction and qRT-PCR analysis

Fresh peel and flesh tissues were harvested and thoroughly ground into powder with liquid nitrogen. The total RNA was isolated from samples using the Omini-Plant RNA Kit (CWBIO, Beijing, China) following the manufacturer's instructions. The quality and integrity of the total RNA was assessed using a spectrophotometer in conjunction with gel electrophoresis analysis as described previously [50]. Approximately, 1.0 μg RNA was used to synthesize the first-strand cDNA using a PrimeScript RT reagent kit with gDNA eraser according to the manufacturer's instructions (Takara, Shiga, Japan). Quantitative real-time PCR (qRT-PCR) was conducted on Bio-Rad CFX96 Real-Time PCR system using specific gene primers that were listed in supplementary Table S1, and a housekeeping gene, *CpTBP1* (accession No. JQ678780), was used as a reference gene to normalize the expression levels of target genes according to previous study [36]. Each experiment was conducted for three biological replicates, and each replicate containing three fruits.

## Statistical analysis

Data analyses and standard error calculations were statistically performed using Microsoft Excel 2016 (Microsoft Inc., USA), and *t*-tests were performed with the SPSS program (v21.0; SPSS Institute, USA). GraphPad Prism (v9.0) and TBtools (v2.0) was used to process the data and draw the figures.

### Supplementary Information

**Additional file 1:** **Fig S1.** The expression levels of *CpMAPKs*in fruit peel in response to ethephonand 1-MCP treatments. The papaya peel tissues were sampled at 0, 3, 6 and 9 d after beingsubjected to ethephon and 1-MCP treatments. The relative expression levels were determined by real-time reverse transcription PCR, and calculated by ΔΔ*C*tmethod. The red asterisks indicate significant differences between CK and 1-MCP treatment, and black asterisks indicate significant differences between CK and ethephon treatment by Student’s t-test: * *P*<0.05; **P<0.01, respectively. **Table S1.** Primers used for qRT-PCR analysis.

## Data Availability

All data supporting the findings of this study are available within the paper and within its supplementary materials published online.
