# Supplementary material for: Genome-wide identification of MAPK family in papaya (Carica papaya) and their involvement in fruit postharvest ripening
Source: BMC Plant Biol. 2024 Jan 24;24:68. doi: 10.1186/s12870-024-04742-0 (PMC10807106; doi:10.1186/s12870-024-04742-0)
Supplement: Supplementary file 1 — Additional file 1: Fig S1. The expression levels of CpMAPKsin fruit peel in response to ethephonand 1-MCP treatments. The papaya peel tissues were sampled at 0, 3, 6 and 9 d after beingsubjected to ethephon and 1-MCP treatments. The relative expression levels were determined by real-time reverse transcription PCR, and calculated by ΔΔCtmethod. The red asterisks indicate significant differences between CK and 1-MCP treatment, and black asterisks indicate significant differences between CK and ethephon treatment by Student’s t-test: * P<0.05; **P<0.01, respectively. Table S1. Primers used for qRT-PCR analysis. [file 12870_2024_4742_MOESM1_ESM.pdf]

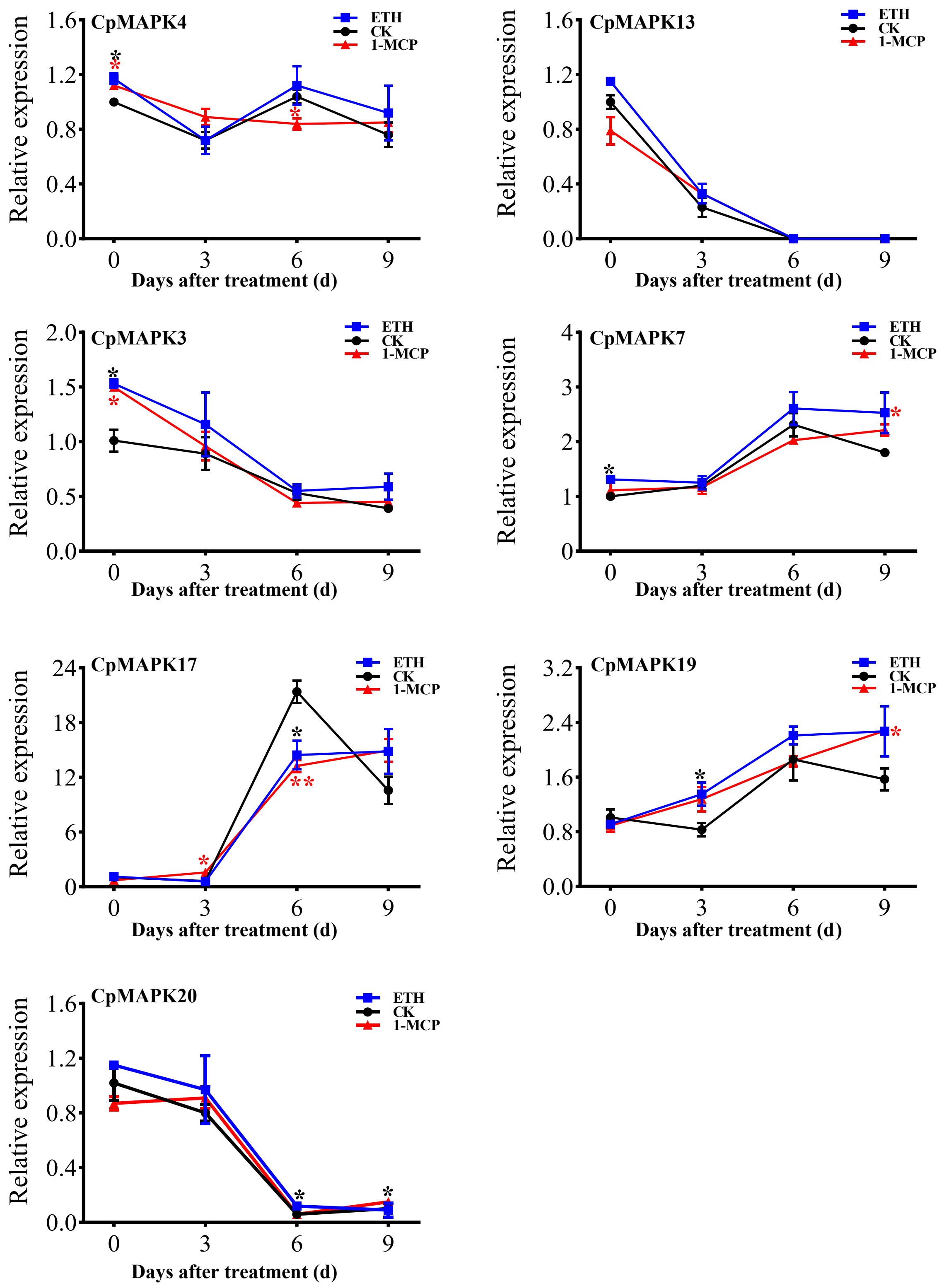

Fig. S1 The expression levels of *CpMAPKs* in fruit peel in response to ethephon and 1-MCP treatments.

The papaya peel tissues were sampled at 0, 3, 6 and 9 d after being subjected to ethephon and 1-MCP treatments. The relative expression levels were determined by real-time reverse transcription PCR, and calculated by $\Delta\Delta C_t$ method. The red asterisks indicate significant differences between CK and 1-MCP treatment, and black asterisks indicate significant differences between CK and ethephon

treatments by Student's *t*-test: * $P<0.05$; ** $P<0.01$, respectively.

Table S1. Primers used for qRT-PCR analysis

Gene name	Forward primer (5'-3')	Reverse primer (5'-3')
CpTBP1	GGTAGTAGTAGTTAGGTATGTG	GGCAATCTGGTCTCACTT
CpMAPK3	CTCGACCAACTGCCGAGAAT	AGATGCAACCGACAGACCAC
CpMAPK4	CCTTGGGTTCCTACGGAGTG	CTCCAGCAAATCAACCGCAC
CpMAPK6	ATACCACCACCTTCAGAGGGA	ACTTCAAACTAAACCCCTGGCA
CpMAPK7	ATGCTGACCCCCTAGCCTTA	GAGGATTGCACCGTGGATCA
CpMAPK9	TTCGAGTCCCAAAACGCAGT	GTCAGAGACTCTTTCCGACCAG
CpMAPK13	CGAAGCAAGAATGGCAGGTG	AACTTGTCCTTCTCTGGCGG
CpMAPK17	TCCCGAATCGCTTTGATGTCT	AAACACGCTCGAAGATGCCA
CpMAPK19	ACGCCAGCCATTGACATTTG	AGGGGTTTTCCTCCGCATTT
CpMAPK20	TGATCCAAAAGACCGGCCAA	TGTCAAGTGGGATCACTGGC
